# GSA Journals Panel With the Editors-in-Chief: Recent Trends, Developments, and Opportunities

**DOI:** 10.1093/geroni/igaf122.1232

**Published:** 2025-12-31

**Authors:** Jessica Kelley, Lewis Lipsitz

## Abstract

The Gerontological Society of America is committed to cultivating excellence in interdisciplinary aging research and education to advance innovations in practice and policy. This session brings together the seven Editors-in-Chief of the GSA journal portfolio, The Journals of Gerontology, Series A: Biological Sciences and Medical Sciences, The Journals of Gerontology, Series B: Psychological Sciences and Social Sciences, Innovation in Aging, The Gerontologist, and Public Policy & Aging Report, to discuss the scope and vision of each journal. Judie Lieu, Vice President for Publishing and Professional Resources, will present an overview of the GSA publishing portfolio and strategic initiatives to continue to publish the top science in aging. Then each Editor will discuss scientific priorities for their journal, upcoming opportunities for publishing, and new trends. The panel will address recent developments in grant funding and science, and how the journals are responding. Ample time for audience questions will be reserved. This session will be useful for emerging scholars seeking to publish in the GSA journals, as well as seasoned scholars who are interested in new developments in GSA publishing priorities and efforts.

